# Indents

**Published:** 1907-01-15

**Authors:** 


### INDENTS.

☞ **Brief Articles of Technical or Scientific Value will receive notice and comment. Contributions invited.**

**Ethics.** I don't think we need any special system of ethics for dentists. We do need, however, more education and training owing to the fact that the moral faculty is not innate and, therefore, subject to the improvements we really desire. The more one knows the less he goes astray: the more informed men are the more they agree together. We must take heed and not allow these hustling, selfish, modern commercial attributes to stain our profession any further.—Alfred Owre, *Dental Review.*

**The Evolution of the Student.** In the evolution of a student in a dental college into a successful practitioner, with something more than a local reputation, there are points to be considered apart from his accomplishments in the way of a high degree of skill based upon accurate scientific knowledge: although this must constantly stand first among all professional men who seek to bring physical comfort to their fellow beings.

The particular point I desire to write about in a few words is one in which the student while at college can give particular attention viz;—To cultivate the ability to speak and write intelligently of what is in his brain. The Articulator stands with open page to every student of the Kansas City Dental College and offers an opportunity which if embraced can readily make intelligent, original authors, or useful commentators.

The various societies of the college also extend to every one an opportunity to become ready speakers upon the various subjects brought before them for discussion. Learn to think and speak, and then when you have anything to say, when in the future you are in larger meetings, (as of course you will be) you will not stammer, — have stage fright, and fail to let your audience know what you mean. This failure is painfully evident in every state or national gathering.

Do not let this come to you Now is the season to prevent such a possibility. Take advantage of it, If you can only say five words, or write only one sentence, do so and soon good speeches and pages of good writing will give you a desirable fame.—J. D. Patterson.

**Suitable Neckwear for Dentists.**

A dentist's work is sufficiently trying without having anything so absurd to encumber him as some of the stiff, high collars that are worn as a tribute to modern style. If he operates all day at the chair whether he sits on a stool, or stands as most operators do—there are times when to get proper vision of his work he must bend or twist his neck. It matters not if he operates through a mirror, which he should learn to do there are still circumstances under which it is necessary for the best results in an operation to bend in such a way as to place the neck under tension if it is bound by a tight-fitting starched collar. It is doubtless expecting too much of dentists to ask them to ignore fashion sufficiently to discard the regulation linen collar while operating, though it would add immeasurably to their comfort and even to their health if they would do so. The evils of the high, stiff and tight collar are not recognized as they should be. Aside from the manifest discomfort there is an element of positive danger in impeding the circulation when the head is bent, causing a congestion in the brain which may work permanent injury.

In watching operators at our public clinics it may frequently be observed that they involuntarily reach the hand up to pull at their collar at intervals during the operation, and if they become greatly absorbed in their work and remain in one position any length of time it will be noticed that the face becomes flushed and the veins stand out over the temples in a wholly abnormal condition.

It is needless to outline the train of evils which may follow this state of affairs if kept up long enough, and it is high time that dentists recognized this danger in their work. While they must not have the courage to wholly abandon the absurd neckwear which modern fashion imposes on suffering humanity, they can at least wear collars that are sufficiently large for the neck to admit of considerable movement without undue impingement on the vessels and nerves, and what will add materially to their comfort they may have the neckbands of their shirts ironed without starch.

A little attention to comfort in dress during the long and arduous hours of operating will not only conduce to health but will actually aid in efficiency of service and it is better that a dentist sacrifice somewhat in style than to jeopardize his health and comfort by a blind following of fashion.—Dr. C. N. Johnson in *Review*.

**Efficiency.** A dentist in order to be properly equipped to build up a successful practice must possess a rare combination of qualities, most of which can only be acquired by continued application.

The chief qualifications necessary to success is without a doubt "Efficiency". In fact it is the corner stone in the foundation of success in dentistry. It is just as important that the dentist be proficient in every department of his work as it is for the surgeon to be an expert operator or for the physician to select the proper therapeutic agent in formulating a prescription.

One of the most serious mistakes the young dentist can make is to get the notion into his head that because he is a graduate of a reputable dental school he is fully equipped to make a sucess of the practice of dentistry and does not require any more knowledge.

The college course is simply a start in the right direction.

It illustrates the proper methods of obtaining knowledge and how to study intelligently. After graduation there is almost certain to be at least a few things which seem exceedingly difficult of accomplishment. It maybe in extending a certain class of cavities for the removal of an inlay matrix; or restoring contour in building up gold filling in certain class of cavities: or in obtaining the maximum of strength and greatest smoothness in a joint between an artificial crown and the root to which it is attached; or it may be some other problem which troubles him a great deal, and of the result of which he is never certain. The proper thing for the dentist to do in any case as soon as he finds that he can not get the best results, is to perfect himself in that class of work.

He should study the literature of the profession and obtain all the knowledge possible on the subject. Then go to some members of the profession who are particularly skillful in this line of work and ask to have it demonstrated to him; or go to work himself on the proposition and not give up until he knows he is proficient. In this age of progress no dentist can afford to stand still. As a Student he should demonstrate to his teachers that he is made of the right kind of stuff, and after graduation he should endeavor to retain the respect of his Alma Mater if he ever expects to gain the respect of the dental profession in general, without which a dentist misses much in life in professional progress.

No man can advance as rapidly plodding along alone as he can by the aid of those interested in the same persuits. In mixing with his professional friends, being ready and willing at all times to put his shoulder to the wheel he will become a better man in many ways, with a distinct personality instead of being a nonentity.

A dentist should aim to rise above the average in efficiency and should always bear in mind the fact that the reason the average is so low in dentistry is because so many members of the profession are content to be average men. The laity are recognizing this fact more and more each day, and in fact the time has arrived when the public will not stand for inferior dental work.—*Odontoblast.*

**Painless Methods.** First let your patients know that you are fully aware of their condition, and position in the matter, but impress upon them that they have your full sympathy, and that you are just as anxious not to hurt them, as they are not to be hurt.

Tell them that in order not to hurt them, you are going to make use of methods and means, which make it possible to do their work free from pain, but that in doing this, you must have their hearty support. Caution them to notify you the moment they feel the slighest pain whatever.

Make no promise to a patient which you do not expect to keep to the letter.

Cultivate a delicate touch. A touch can be cultivated which is both delicate and firm. A touch so light that it fails to irritate, and yet so full of strength, and character, that it inspires confidence.

In preliminary examinations if a cavity is found, that is enough. The knowledge that this cavity exists should suffice. Defer a complete examination as to the extent of that cavity, until you have made an appointment to care for that particular case, and have plenty of time to investigate and treat existing conditions.

If a patient is suffering or has been suffering, do not go probing until youhav e obtainedthe history of the case. Gain, from information furnished, and idea of what to expect, whether sensitive dentine, pulpitis. or peri-cementitis. Then proceed in the most cautious manner to confirm this history and finding a condition, treat it, using every precaution not to hurt.

If the rubber is to be applied, and there is the the least likelihood of causing the slighest pain in so doing have your assistant bathe the gums for a few moments with a solution of cocaine in Adnephrin and water. If there is still danger of considerable difficulty in placing the rubber cloth, have the time lengthened,and the cocaine solution used until the gums show a blanching.

Then proceed with the utmost care not to cause pain in adjusting the rubber. This is of the greatest importance in many cases. Many times have I seen patients in such a nervous state by the time the dam was in place that they were in no condition to be operated upon.

Having the rubber in place dry away moisture, being careful not to cause pain in so doing.

Cautiously open up the cavity and remove all debris, using sharp chisles and excavators resorting to the use of the engine and bur as little as possible until you have ascertained the kind, nature and sensitiveness of the cavity.

The dental engine is one of the greatest bug-bears to our patients, and should be used only when and where it is absolutely needed, and always when and where it can be used without causing pain.

In learning to practice humanitarian methods time must not be considered. Results are what we are after. When we become skilled in producing results, then it is permissible to consider the question of time and the saving of same.
W. G. Ebersole, in *Dentists Magazine*

**Original Method of Making Gold Inlay.**

As before mentioned there are several methods of procedure in producing the gold inlay, with varying results. I pin my faith to the pure gold inlay with the platinum matrix and the method of making the same is one which I have worked out for myself, and I hope it may be tried at least in order that its easy accomplishment, after a little familiarity with its working, may give to those who use it the same relief from that intense pain with which we are all so familiar that it has given me. It is impossible in this short paper to thoroughly describe the preparation of cavities. It is sufficient to say that there are two important mechanical principles to be observed.

FIRST; that the cavity preparation must provide a sufficient draft to provide for the easy withdrawal of the matrix from the cavity.

SECOND; sufficient anchorage must be made to withstand the strain of mastication, and provide firm retention of the inlay within the cavity. When the first is not possible cement may be used to fill deep undercuts or pits caused by decay.

The cavity being prepared, use platinum foil one and one-half thousand gauge, or fifteen ten-thousandths. This gives a matrix with much more stiffness and rigidity than can be made by the one one-thousandths gauge. Cut the piece large enough to cover the bottom of the cavity and extend well over the margins. Now have suitably-prepared sticks, pine, or other wood six or eight inches in length, shaped at one end to fit the cavity, gently force platinum into the cavity with the stick, rubbing the same over all surfaces, allowing the foil to fold over margins if necessary

Burnish well into place, and over margins. Remove matrix and anneal, replacing same in cavity, and do as before. Do this until the matrix is fairly well in place. Now, take uncured red label rubber, which is the rubber used to label the ordinary rubber garden hose, and cut piece large enough to fill cavity, and much more, then direct patient to close teeth upon same, forcing down upon it for a few minutes and kneading the rubber between the teeth, care being taken to caution the patient never to remove the teeth from contract with the rubber, as the matrix may be withdrawn and spoilt. Have patient carefully open the jaws, holding your finger upon the rubber and remove rubber and matrix. The matrix maybe broken in places, but if this is not over the margins no harm is done. Place matrix on charcoal block, and have pure gold plate cut into pieces, two or three of which place in matrix: if matrix is broken in body of cavity melt one or two pieces of gold into a ball and place same over break, sweating same into matrix: build up piece by piece but do not at any time use large flame, as the secret of success is in sweating the gold into place, otherwise you will flow your gold over the outside.

The inlay unfinished may be tried in the cavity at any time to determine the exact amount of gold required. No investment is used; nothing to prevent the gold from flowing on reverse side of matrix or elsewhere: no borax is used except at times as in sweating you may nor flow your gold sufficiently well over margins, in which case a toothpick is dipped in vaseline borax and a film is spread over margin desired to be covered: very small pieces of gold may be used for this purpose. If you have followed the technique you will have a gold inlay about the exact shape desired. The inlay being of pure gold, its margins maybe burnished into place before and after setting a very strong point in favor of the pure metals.—Dr. H. Barnes, *Summary*.

